# Financial Losses Arising from Cattle Organ and Carcass Condemnation at Lokoloko Abattoir in Wau, South Sudan

**DOI:** 10.1155/2023/7975876

**Published:** 2023-03-20

**Authors:** Alfateh Taha, Shereen Saad, Ambros Jubara, Charles Wani, A. M. Phiri, Martin Simuunza, Musso Munyeme, Bernard Hang'ombe, Chisoni Mumba

**Affiliations:** ^1^Department of Clinical Studies, University of Bahr El Ghazal, College of Veterinary Science, Wau, South Sudan; ^2^Department of Clinical Studies, School of Veterinary Medicine, University of Zambia, Lusaka, Zambia; ^3^Department of Disease Control, School of Veterinary Medicine, University of Zambia, P.O. Box 32379, Lusaka, Zambia; ^4^Department of Paraclinical Studies, School of Veterinary Medicine, University of Zambia, Lusaka, Zambia

## Abstract

Slaughterhouses in South Sudan mirror the economic losses resulting from cattle organs and carcass condemnation due to zoonotic and epizootic diseases of livestock, such as tuberculosis, cysticercosis, and hydatidosis in cattle. However, due to the war, slaughterhouse record keeping has been inconsistent in South Sudan, and thus the estimation of diseases in cattle and their impact may be underestimated. Therefore, this study was conducted to estimate the major causes of carcasses and organ condemnation of cattle slaughtered at Lokoloko abattoir and the resulting financial losses. A cross-sectional active abattoir survey involving antemortem and postmortem examinations was conducted on 310 cattle between January 2021 and March 2021. Furthermore, five-year (September 2015–September 2020) retrospective data on meat inspection records were also collected and analyzed. During the antemortem inspection of the active abattoir survey, 103 (33.2%) cattle had signs of disease. These signs included herniam 17 (5.5%), local swelling 16 (5.2%), lameness 15 (4.8%), emaciation 13 (4.2%), blindness 12 (3.9%), depression 11 (3.5%), pale mucus membrane 7 (2.3%), nasal discharge 5 (1.6%), lacrimation 4 (1.3%), and salivation 03 (0.97%). Postmortem inspection revealed gross pathological findings on 180 (58.6%) carcasses, out of which 47 (26.1%) livers and 31 (17.2%) hearts were condemned due to various causes. The active abattoir survey and the retrospective data revealed that tuberculosis, fascioliasis, hydatidosis, and heart cysticercosis were the leading causes of condemnation of carcasses and organs. In the active abattoir survey, a total of 19,592,508 South Sudanese Pounds, equivalent to US$29,686 was lost from organ condemnation, while in the retrospective data; the overall direct financial loss during the five years was estimated to be 299,225,807 South Sudanese Pounds equivalent to US$453,372. This study revealed that bacterial and parasitic diseases were the common causes of carcass and organ condemnations and caused significant financial losses at Lokoloko abattoir in Wau, South Sudan. Therefore, there is a need for training farmers on cattle disease management, heightened meat inspections, and proper disposal of condemned meat.

## 1. Introduction

Cattle diseases are considered a major problem and cause significant financial losses in countries where livestock production is an important sector of agricultural practice [1]. The developing countries have about two-thirds of the world's livestock population, but their meat and milk production is less than a third of the world's [2]. This is due to many constraints that hinder the potential of livestock production, such as a lack of appropriate disease control policies and poor veterinary services [3]. Among these countries, South Sudan has a large livestock population in Africa, with an estimated population of 49 million cattle [4]. Today, South Sudan's ruminant livestock wealth is still largely in the hands of traditional agropastoralist and pastoralist systems that hold 47% and 43% of South Sudan's livestock wealth, respectively [5]. The remaining 10% are in the hands of smallholder livestock keepers, mainly in urban and periurban areas [6]. As mentioned earlier, the constraints lead to significant annual economic losses mainly due to the condemnation of edible animal organs and carcasses from different country abattoirs [7].

Abattoirs continue to be the source of valuable data for the epidemiology of cattle diseases. They also provide information on the extent to which a community is exposed to certain zoonotic diseases [8]. They can also estimate the financial losses incurred through the condemnation of affected organs and carcasses [8]. Postmortem examinations of animals slaughtered in abattoirs give a better chance of recognizing the most important prevalent diseases [9, 10]. The source of the animal from an infected region must be identified to avoid introducing clinically diseased animals into the abattoir. The postmortem examination should be conducted within 24 hours of slaughtering the animal and must be repeated when there is a suspected case [11].

Meat inspection and hygiene of food animals constitute a potential source of data and information for epidemiological studies and preventive veterinary medicine [12]. Meat-borne diseases create a human health hazard and cause substantial economic losses in South Sudan [13]. A retrospective study on meat condemnation is imperative for veterinary public health to establish a control strategy for meat-borne diseases.

Diseases are the main reason beef gets condemned in abattoirs [14]. Studies in South Africa, Ethiopia, and Zambia have shown that a large quantity of meat (carcass/organ) was totally or partially condemned due to zoonotic diseases such as tuberculosis, fasciolosis, hydatidosis, and cysticercosis [15, 16]. Therefore, the current study investigated the major causes of carcass and organ condemnation and their financial losses in cattle slaughtered at Lokoloko abattoir in South Sudan.

## 2. Results

### 2.1. Abnormalities Observed during Antemortem Examinations (Active Abattoir Survey)

Out of 310 cattle subjected to antemortem inspection, showing abnormalities and signs of disease, 103 (33.2%) cattle had various types of disease signs including hernia 17 (5.5%), local swelling 16 (5.2%), lameness 15 (4.8%), emaciation 13 (4.2%), blindness 12 (3.9%), depression 11 (3.5%), pale mucus membrane 7 (2.3%), nasal discharge 5 (1.6%), lacrimation 4 (1.3%), and salivation 03 (0.97%), as shown in (Table 1).

### 2.2. Postmortem Examination

The postmortem examination results revealed that 180 (58.6%) carcasses, 47(26.1%) livers, and 31 (17.2%) hearts were condemned due to various causes. Tuberculosis (26.1%) and fascioliasis (25.6%) were the leading causes of liver condemnation, followed by hydatid cyst (31.1%) and heart cysticercosis (17.2%), respectively. Assessment of the financial loss indicated that losses due to tuberculosis were the highest at 8,069,148 South Sudanese Pound, equivalent to about US$12,226, followed by fascioliasis with a financial loss of 6,331,440 South Sudanese Pounds (US$9,593), as shown in Table 2.

### 2.3. Retrospective Abattoir Study (2015–2020)

The retrospective data showed that the whole carcass, the liver (fascioliasis/hydatidosis), and the heart were the most condemned organs, with condemnation rates of 31.7%, 21.8%, and 24.8%, respectively. The major causes of organ condemnation were generalized tuberculosis, liver fascioliasis, liver hydatidosis, and heart cysticercosis. Assessment of the financial loss indicated that tuberculosis was associated with the highest financial losses at 122,881,465 South Sudanese Pounds (US$186,184), followed by fascioliasis with financial losses of 117,828,060 South Sudanese Pounds ($US178,527) as shown in Table 3.

### 2.4. Discussion

The present study revealed that carcasses, livers, and hearts were condemned due to various causes. Tuberculosis was the cause of the whole carcass condemnations. Fascioliasis was the leading cause of liver condemnation, followed by hydatidosis and heart cysticercosis. This result is comparable with Ethiopian [17] and South African [18] studies. The retrospective data showed that the liver and heart were the most condemned organs, with a condemnation rate of 16.9%, 16.8%, 22.4%, and 19.2%, respectively. The major causes of organ condemnation were generalized tuberculosis (24.6%), liver fascioliasis (16.9%), liver hydatidosis (16.8%), and heart cysticercosis (19.2%), respectively.

The active survey shows that the liver was the most condemned organ primarily due to parasites (56.7%), which include *Fasciola* species and cystic echinococcosis. However, the retrospective data show a lower condemnation rate of 33.7%. The lower rate could be due to missing reports during postmortem examination. Postmortem examination of the animal carcass and organs showed that the whole carcass condemnation was due to generalized tuberculosis with a financial loss of US$198,410 and 54,589 kg weight loss from retrospective and active surveys, respectively. The prevalence of tuberculosis in the active survey was 15.2%, which was slightly higher than the 13.7% reported in the retrospective survey. This difference in the two surveys could be due to the limitation of meat inspection in identifying tuberculosis and missing reports in the retrospective study [19].

From the retrospective and active abattoir surveys in the present study, a total of 5,862 cattle whole carcasses were condemned due to generalized tuberculosis and heart cysticercosis. These conditions are known to be major causes for the condemnation of carcasses in many African countries such as Ethiopia [20], Sudan [3, 18], South Africa, and South Sudan [13]. The prevalence of liver fascioliasis reported in the present study, both in retrospective and active data surveys, was 9.4% and 14.8%, respectively. These findings are similar to those reported in a study conducted in Tanzania, which estimated liver fascioliasis prevalence at 17.8% [21]. The overall prevalence of fascioliasis obtained in the present study (23.9%) is also like the 22.9% reported in the previous study from Ethiopia and 20.77% reported in Zambia [11, 16, 22].

During the active abattoir survey and retrospective study, 14,101 cattle whole carcasses were condemned due to tuberculosis, fascioliasis, hydatidosis, cysticercosis bovis, and pneumonia [23]. These conditions were known to be major causes for the condemnation of carcasses in many parts of Ethiopia [14, 24]. Underreporting during routine meat inspection could have affected carcass condemnation due to tuberculosis, and/or tuberculosis-like lesions could be more than what had been recorded in this study [17, 25]. From the active abattoir survey, the overall economic loss incurred due to carcass and organ condemnation was 19,592,508 South Sudanese pounds, equivalent to US$29,686. However, the retrospective study reported a loss of 299,225,807 South Sudanese pounds, equivalent to US$453,372. This loss could be much higher if the indirect loss is computed and incorporated. However, other African countries such as Nigeria and Rwanda reported losses of US$18200 and US$4810, respectively [26].

## 3. Materials and Methods

### 3.1. Study Site

**Figure 1:**
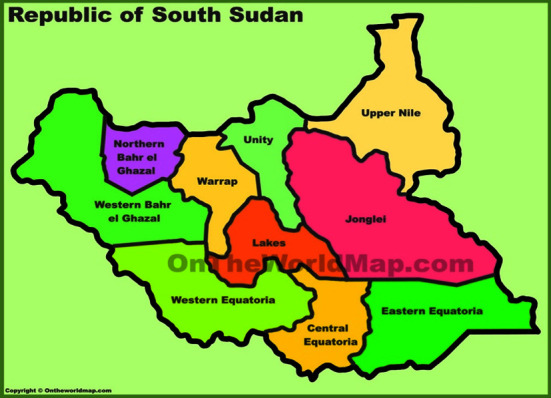
Map of the study area.

The study was conducted (Figure 1) in Wau Town, Western Bahr el Ghazal state, South Sudan. The state shares international borders with Sudan to the North and Central African Republic to the West, with coordinates of 8.6452°N, 25.2838°E, and 626.9 meters above sea level. The climate is tropical, with annual rainfall between 400 and 1600 mm and a temperature between 23.8°C and 40°C. Wau is located on the western bank of the Jur River, and it consists of two administrative parts: Wau North and Wau South. North and South Wau have their abattoirs beside small slabs where the condemned carcasses and organs are incinerated briefly. A slab is a slaughter space where the facility will be slowly transformed into an economical, low-throughput slaughter slab after an initial fitting of a gantry hoist, concrete slab, and metal roof. The slab must contain a floor ring to hold and grasp castles, skinning frameworks for cattle and small stock, rails for hanging the carcasses, and an adequate and convenient water supply.

There are two types of South Sudanese cattle: Baqqara and Nilotic. Food and Agriculture Administration (FAO) estimated report, South Sudan's cattle population at 12 million and 151,320 people in 2008. Wau Town was chosen for this study because it is the largest town in the greater Bahr el Ghazal region, the hub for livestock marketing and has the largest abattoir with experienced veterinarians (Figure 1).

### 3.2. Study Population

The study animals were cattle brought to the abattoir for slaughter from different counties of Western Bahr el Ghazal State and neighbouring states.

### 3.3. Study Design

This was a cross-sectional study design involving an active and retrospective abattoir survey.

#### 3.3.1. Active Abattoir Survey

A cross-sectional study was conducted from January 2021 to March 2021 to identify the major causes of whole carcass and organ condemnation and estimate the direct economic losses from organ condemnation in cattle slaughtered at Lokoloko abattoir. A total of 310 cattle were examined using antemortem and postmortem examinations. The study animals were selected using a simple random sampling method. Only the Lokoloko abattoir was chosen because it is the only abattoir in the study area handling many cattle for slaughter besides small slabs.

#### 3.3.2. Ante- and Postmortem Examination

Before the examination, animals were given special marks for identification at postmortem. General behaviour, signs of disease, nutritional status, cleanliness, and any abnormalities were recorded as described by Nigo et al. [10]. Judgment was also done based on the procedure described by [10] and the legal framework found at https://www.fsis.usda.gov/. Postmortem examination was conducted through visual inspection, palpation, and systematic incision of each visceral organ, particularly the liver, lung, heart, and whole carcass, for the presence of cysts, parasites, and other pathological lesions described by Animal Disposition/Food Safety: Postmortem Inspection 3/03/19.

#### 3.3.3. Retrospective Abattoir Study

A retrospective study was conducted using the postmortem meat inspection records of the abattoir from September 2015 to September 2020. Data were obtained with the help of an experienced team of veterinarians from the Ministry of Animal Resource. Information collected included the number of cattle slaughtered, the type and number of condemned organs, carcasses, and causes for each condemnation.

### 3.4. Analysis of Financial Loss due to Total or Partial Organs Condemnations

Budgetary analysis was used to estimate total annual financial losses incurred due to whole carcasses and partial condemnations for each year from 2015 to 2020 using several parameters, which included the following: (1) The average number of cattle slaughtered per year. (2) Prevalence of most affected organs per year. (3) The average sale price (Av Pr) of the most affected organ each year was obtained through a survey conducted at the Wau abattoir (Lokoloko). (4) Average weight in kilograms (Av Wt.) of most affected organs in mature cattle. The direct loss (DL) resulting from condemnations of whole and partial condemnation was calculated using the formula below (as described by Swai and Ulicky, 2009):

(1) $$\begin{array}{c} DL=TNA\times Pf\times \left(Ao\;Wt\right)\times \left(Ao\;\;\mathrm{Pr}\right)\text{,} \end{array}$$

where TNA = total number of cattle slaughtered, Pf = prevalence of most affected disease, Ao Wt = average weight of affected organ (kg), and Ao Pr = average price of affected organ/kg.

### 3.5. Data Collection Tool

A questionnaire and sample checklist were developed and applied for active cross-sectional and retrospective studies.

### 3.6. Statistical Analysis

Collected data were entered and stored in Microsoft excel and analyzed using Statistical Package for Service Solutions (SPSS) version 24 (IBM, USA). Descriptive statistics were used to determine organ condemnation, defined as the proportion of condemned organs to the total number of organs examined.

### 3.7. Condemnation Compensate

Compensation for the losses was at half rate and was done by the government and NGOs. The condemned carcasses and organs were incinerated.

## 4. Conclusion

Bacterial and parasitic diseases were the common causes of carcasses and organ condemnations. The most condemned organs were the liver and heart. The major causes of partial or total organ condemnation were mainlyfasciolosis, hydatidosis, and cysticercosis. Furthermore, the major cause of carcass condemnation was tuberculosis. These diseases and pathological conditions resulted in considerable financial loss in the Lokoloko abattoir in Wau, South Sudan. Cattle keepers are responsible for the health of their cattle. The government should provide all the possible preventive measures against cattle diseases. This is necessary if a disease is exceptionally infectious or dangerous. Cattle farmers must ensure proper hygiene at their farm and alert and inform the government in case of symptoms of a disease show up. The government should educate the cattle keepers (farmers) to report (suspected) animal diseases for the response. Both cattle farmers and the government should employ routine cattle vaccination. Moreover, strengthening meat inspection at the abattoir is highly recommended.

## Figures and Tables

**Table 1:** Abnormalities observed during antemortem examinations.

Abnormalities	Cases number	Percentage (%)
Hernia	17	5.5
Local swelling	16	5.2
Lameness	15	4.8
Emaciation	13	4.2
Blindness	12	3.9
Depression	11	3.5
Pale mucus membrane	07	2.3
Nasal discharge	05	16
Lacrimation	04	1.3
Salivation	03	0.97
Total	103	33.2

**Table 2:** Cases and financial loss from cattle total and partial condemnation in Wau abattoir.

Case or disease	Mood of condemnations	Average no. of cattle slaughtered	Average no. of lesions	Average prevalence (%)	Price SSP (kg)	Average weight condemned (kg)	Cost (SSP)	(USD) 1$ = 660ssp
Generalized tuberculosis	Total	310	47	15.2	05	2259	8,069,148	12,226
Liver fascioliasis	Partial	310	46	14.8	06	1550	6,331,440	9,593
Liver hydatidosis	Partial	310	56	14.8	06	850	4,075,920	6,176
Heart cysticercosis	Partial	310	31	10	05	720	1,116,000	1,691
Total							19,592,508	29,686

*Note.* 1 US$ = 660 SSP by the time of the data collection January and February 2021 (Central Bank 2021).

**Table 3:** Cases and financial loss due to cattle total and partial condemnation from December 2015 to December 2020 in Wau abattoir.

Case or disease	Type of condemnations	Average no. of cattle slaughtered	Average animals with lesions. suggestion	Average prevalence	Price (kg)	Average weight condemned (kg)	Cost in SSP	Cost in USD 1$ = 660ssp
Tuberculosis	Total	10,800	3480	13.7	05	523000	122881465	186184
Liver fascioliasis	Partial	10,800	2350	9.4	06	889,000	117,828,060	178,527
Liver hydatidosis	Partial	10,800	2340	9.4	06	702000	31,990,982	48,471
Heart cysticercosis	Partial	10,800	2680	10.7	05	185000	26,525,300	40,190
Total			10800				229,225,807	453,372

## Data Availability

All the data generated or analyzed during this study are included in this published article (and its supplementary information files). The datasets generated during and/or analyzed during the current study are available from the corresponding author on reasonable request.

## Supplementary Materials

Supplementary Materials Supplementary Material 1: conceptual framework describes cattle disease's effect and financial consequences.
