# Supplementary material for: Financial Losses Arising from Cattle Organ and Carcass Condemnation at Lokoloko Abattoir in Wau, South Sudan
Source: Adv Prev Med. 2023 Mar 20;2023:7975876. doi: 10.1155/2023/7975876 (PMC10042638; doi:10.1155/2023/7975876)
Supplement: Supplementary Materials — Supplementary Material 1: conceptual framework describes cattle disease's effect and financial consequences. [file 7975876.f1.docx]

**Supplementary Material**

**Supplementary Material 1 Conceptual framework**

consequences.

Financial loss

Due to condemnation of organs/ carcass and treatment costs

Loss of Humans resources

Abattoir

-Total condemnation

-Partial condemnation

Infection of Humans

Direct contact with infected carcass/organ

Lead to environment contamination (Abettor)

Infected cattle

Zoonotic Diseases

Diseases of Economic Importance

**Description:**

This framework conceptualizes the health and economic impact of zoonotic diseases in cattle intended for slaughter in the abattoir. Infected cattle in the abattoir might lead into contamination of the environment before the condemnation process. The abattoir workers might be infected through direct contact with infected carcass/ organs or indirectly via the contaminated environment which will have an impact on human resources (e.g. life loss, disability, absence due to diseases ...Etc). Both infection of humans and condemnation of infected organs/carcasses will lead into a financial loss for cattle owners and abattoir workers.
